# Mitochondria‐Targeted Delivery of siRNA by Amphiphilic Nanovectors Self‐Assembled from a Cationic [4]Helicene‐Squalene Ester

**DOI:** 10.1002/cbic.202500537

**Published:** 2025-10-07

**Authors:** Lamiaa M. A. Ali, Dan–Dan Su, Rebecca Mucci, Kévin Martin, Mihail Barboiu, Jérôme Lacour, Nadir Bettache, Sébastien Ulrich

**Affiliations:** ^1^ Institut des Biomolécules Max Mousseron (IBMM) CNRS ENSCM Université of Montpellier Montpellier France; ^2^ Institut Européen des Membranes Adaptive Supramolecular Nanosystems Group ENSCM CNRS University of Montpellier Place Eugène Bataillon CC 047 Montpellier F‐34095 France; ^3^ Department of Organic Chemistry University of Geneva Quai Ernest‐Ansermet 30 Genève 4 1211 Switzerland

**Keywords:** amphiphiles, helicene, mitochondria targeting, nano‐assembly, RNA delivery

## Abstract

The active delivery of therapeutic nucleic acids with subcellular precision is an open challenge. Herein, a small conjugate molecule, combining a cationic [4]helicene moiety with a lipophilic squalene tail, yields amphiphilic nanoassemblies capable of effectively complexing siRNA within the mitochondria. A proof‐of‐concept in HCT 116 colorectal cancer cells was evidenced by Western blot for the delivery of siRNA silencing the protein expression of the mitochondria‐encoded cytochrome c oxidase subunit 1 gene.

## Graphical abstract

A small amphiphilic helicene effectively complexes siRNA, leading to nanoassemblies which transport siRNA to the mitochondria where it silences the protein expression of the mitochondria‐encoded cytochrome c oxidase subunit 1 gene.

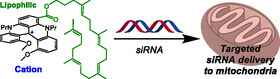

## 1 Introduction

The active delivery of nucleic acid therapeutics requires the use of a carrier which binds, transports, and releases its cargo to the targeted tissue and/or cellular localization. While DNA‐based drugs need to be delivered to the cell nuclei where they will interfere with the replication machinery, RNA‐based drugs such as short interfering RNA (siRNA) or messenger RNA (mRNA) have to be delivered into the cytosol where the translation from RNA to proteins takes place. The targeted delivery of RNA therapeutics to subcellular compartments has hitherto received little attention despite early reports showing for instance that RNA interference (RNAi) was optimal with siRNA located in perinuclear regions.^[^ ^1^ ^]^

Often represented as the energy factory of the eukaryote cells, mitochondria are attracting interest as a pharmacological target.^[^ ^2^ ^,^ ^3^ ^]^ Mitochondria are primary regulators of cellular homeostasis due to their involvement in numerous fateful cellular functions including metabolism, intracellular signaling, immunity, and apoptosis.^[^ ^4^ ^]^ Metabolic plasticity and the proper functioning of mitochondria play a key role in tumorigenesis and in response to anticancer treatments, making mitochondria a promising target for the development of new anticancer therapies.^[^ ^5^, ^6^, ^7^ ^–^ ^8^ ^]^ For instance, the release of cytochrome c from mitochondria to the cytosol activates caspase signaling and triggers apoptosis.^[^ ^9^ ^]^ A window of opportunity would therefore consists in using nucleic acids to perform mitochondrial gene therapy.^[^ ^10^, ^11^ ^–^ ^12^ ^]^ Only a few studies explored siRNA delivery into mitochondria, using the mitochondria‐targeting features of the lipophilic cationic triphenylphosphonium group,^[^ ^13^ ^,^ ^14^ ^]^ cationic peptides,^[^ ^15^ ^,^ ^16^ ^]^ or liposomal nanocarriers.^[^ ^17^ ^]^ Indeed, the existence and importance of the silencing machinery within mitochondria is still a debate and the development of mitochondria‐targeting tools is therefore needed for both fundamental and applied interests. Supporting the idea of RNAi within mitochondria, it was reported that transfected siRNAs are able to enter the mitochondria matrix and specifically silence their targeted mitochondrial mRNAs, downregulating the mitochondria‐encoded cytochrome c oxidase subunit 1 (MTCO1).^[^ ^13^ ^,^ ^18^ ^]^ MTCO is a heme protein present in the mitochondria which plays an important role in the cellular respiratory chain in general,^[^ ^19^ ^]^ as well as in colorectal cancers.^[^ ^20^ ^,^ ^21^ ^]^

**Figure 1:**
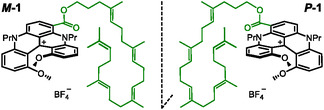
Molecular structures of the two enantiomers ***M*‐1** and ***P*‐1** of cationic [4]helicene–squalene compound **1**. ***Rac*‐1** corresponds to the racemic mixture of compound **1**.

Conjugated fluorophores are interesting probes to combine mitochondrial targeting and imaging.^[^ ^22^ ^]^ In this line, [4]helicene–squalene lipophilic cations were previously developed and showed that they form stable fluorescent nanoparticles, around 100–130 nm in diameter in aqueous media and activatable with red light (*λ*_ex_ = 590 nm, *λ*_em_ = 650 nm).^[^ ^23^ ^]^ These derivatives were also found to rapidly internalize in both U87 MG and PC3 cancer cell lines, accumulating quickly and effectively in mitochondria as revealed by costaining with the MitoTracker dye.^[^ ^23^ ^]^ Since cationic helicenes^[^ ^24^ ^]^ can both interact with nucleic acids and serve as optical probes,^[^ ^25^ ^,^ ^26^ ^]^ we decided to explore the ability of their nanoassemblies to complex siRNA. Herein, we report that compound **1** (**Figure** **1**) effectively leads to nanoparticle formation with siRNA, showing selective delivery in mitochondria and eliciting a silencing effect with siMTCO1 in HCT 116 colorectal cancer cells.

## 2 Results and Discussion

### 2.1 Agarose Gel Shift Assay and Physicochemical Characterization

**Figure 2:**
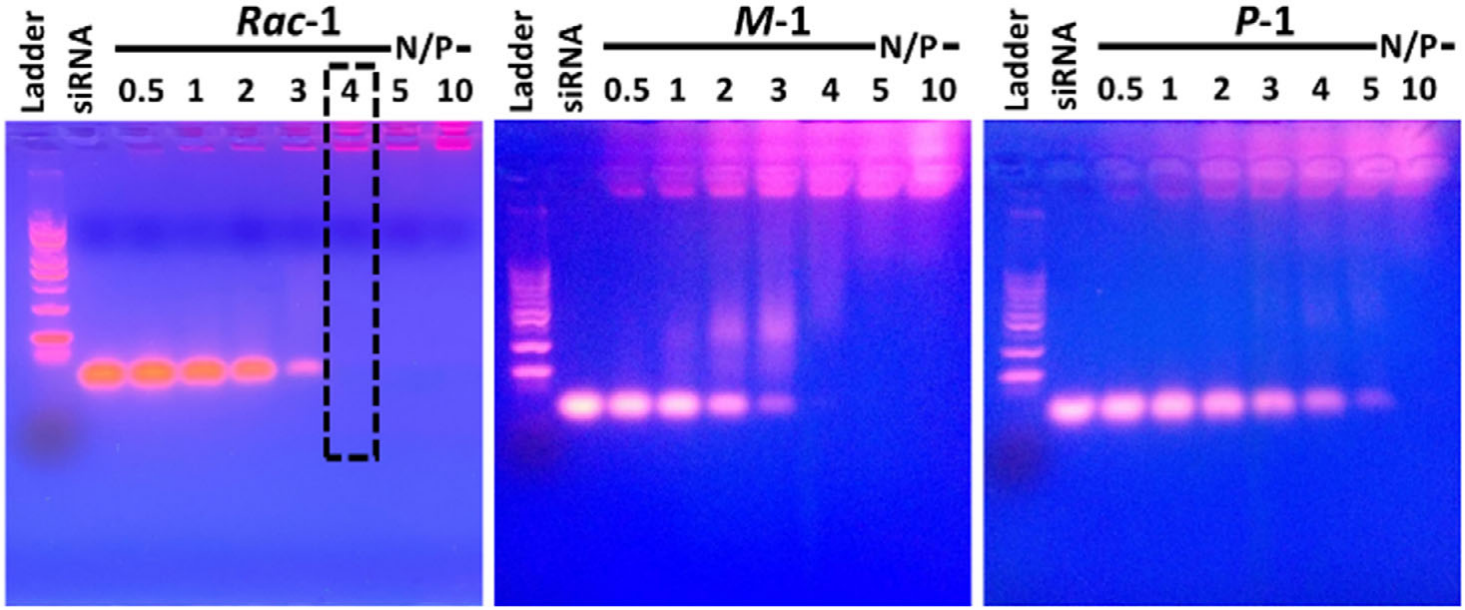
The complexation of siRNA by compounds ***Rac*‐1**, ***M*‐1**, and ***P*‐1** at different N/P ratios assessed by electrophoretic mobility shift assay on agarose gels.

The ability of compound **1** to complex to siRNA was assessed using an electrophoretic mobility shift assay on agarose gel, varying the relative stoichiometry given by the N/P ratio which refers to the ratio of positive charges contained in the lipophilic cation **1** *per* negative charges in the siRNA. ***Rac*‐1** showed a complete complexation of siRNA starting at N/P = 4, as revealed by the complete disappearance of the free siRNA band (**Figure** **2**). The enantiomers ***M*‐1** and ***P*‐1** confirmed the same trend, the chiral preference imposed by the structure of siRNA making ***M*‐1** more potent than ***P*‐1**. For the latter, only partial complexation of siRNA was achieved at N/P = 5—the complete complexation requiring N/P = 10 (Figure 2). This is in agreement with earlier results on cationic [4]helicene derivatives lacking the squalene tail which were shown to bind double‐stranded DNA by intercalation and/or groove binding and display the same superiority of the ***M*** over the ***P*** enantiomer.^[^ ^27^ ^]^

DLS analyses and *ζ* potential measurements for the ***Rac*‐1**–siRNA complexes showed the formation of nanoparticles with a size of 109–179 nm, in line with the nanoassemblies formed in the absence of siRNA that are around 100–130 nm in diameter (**Table** **1**).^[^ ^23^ ^]^ The size and polydispersity steadily decreased with increasing N/P ratio, meaning a higher degree of compaction is reached in the presence of the more lipophilic cation **1**. Similarly, *ζ* potentials increased with the N/P ratio, from –4 mV at N/P = 5 to + 32 mV at N/P = 20, coming to a close value of the nanoassembly of ***Rac*‐1** alone of + 37 mV (Table 1).^[^ ^23^ ^]^

**Table 1:** Dynamic light scattering and *ζ*‐potential measurements of ***Rac*‐1**–siRNA complex at different N/P.

*Rac‐1*–siRNA	Size [nm]	PDI	*ζ* potential [mV]
N/P = 5	178.6 ± 40.8	0.32 ± 0.08	−3.9 ± 11.7
N/P = 10	159.9 ± 49.3	0.32 ± 0.07	12.1 ± 0.8
N/P = 20	109.0 ± 9.6	0.24 ± 0.06	32.4 ± 0.8

### 2.2 Cytotoxicity

**Figure 3:**
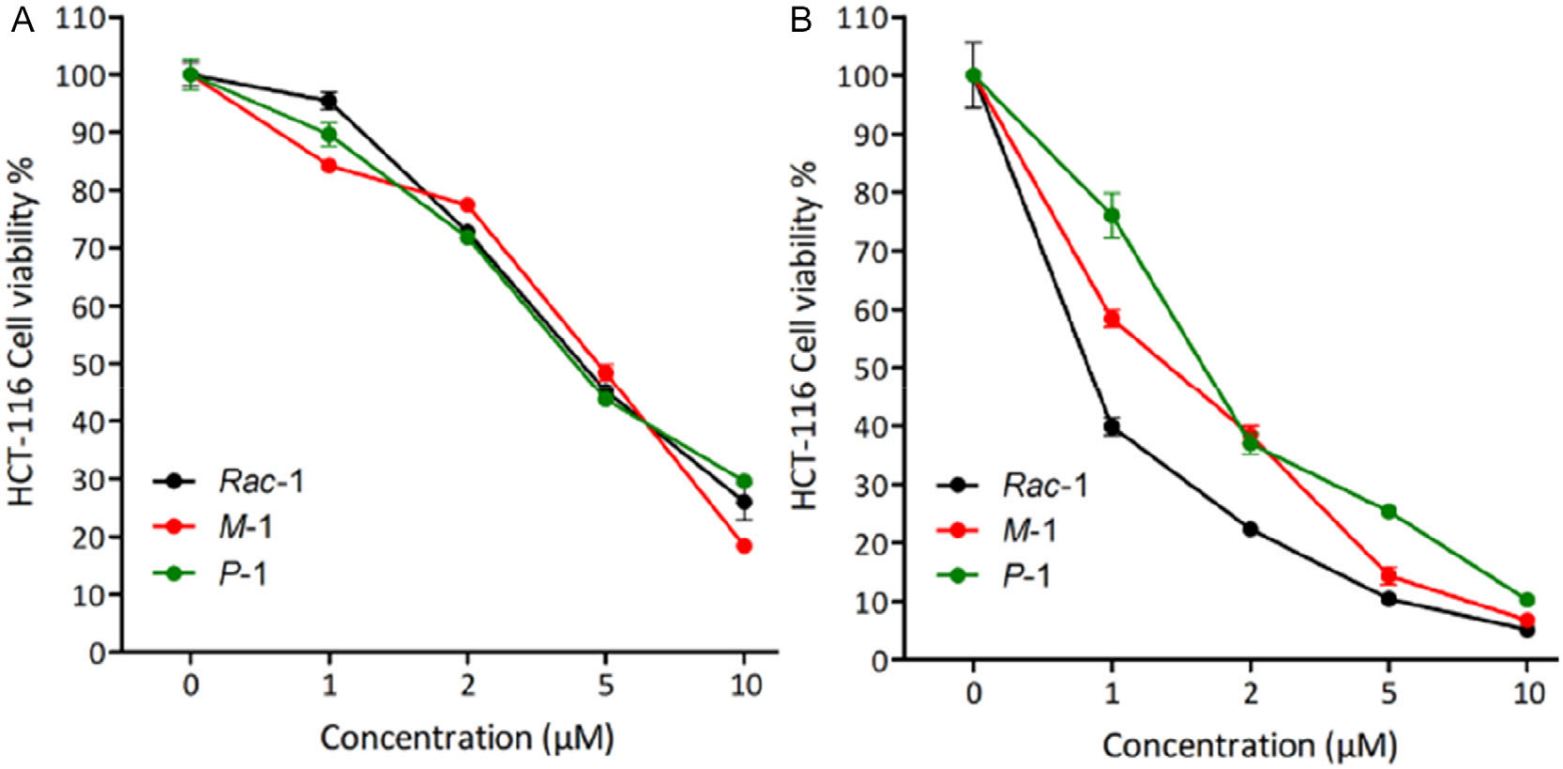
Cytotoxicity study of lipophilic cation ***Rac*‐1**, ***M*‐1**, and ***P*‐1** on human colorectal cancer cells (HCT 116) after A) 24 hr and B) 48 hr incubation. The results are presented as mean ± SEM of three independent experiments.

Before moving to siRNA delivery studies, the cytotoxicity was determined using an MTT assay on human colorectal cancer cells (HCT 116) as previously reported.^[^ ^28^, ^29^, ^30^, ^31^ ^–^ ^32^ ^]^ Since cytotoxicity was previously observed on HeLa cells after 24 hr incubation of a 10 µM dose of the nanoassembly of ***Rac*‐1** in the absence of siRNA,^[^ ^23^ ^]^ we explored lower doses and varied the incubation time. The results revealed a time‐dependent and concentration‐dependent cytotoxicity profile (**Figure** **3**). From this basis, we selected doses lower than 5 µM and incubation time of 24 hr for the following work. The cytotoxicity profile of the individual enantiomers ***M*‐1** and ***P*‐1** followed the same trend (Figure 3).

### 2.3 siRNA Delivery

**Figure 4:**
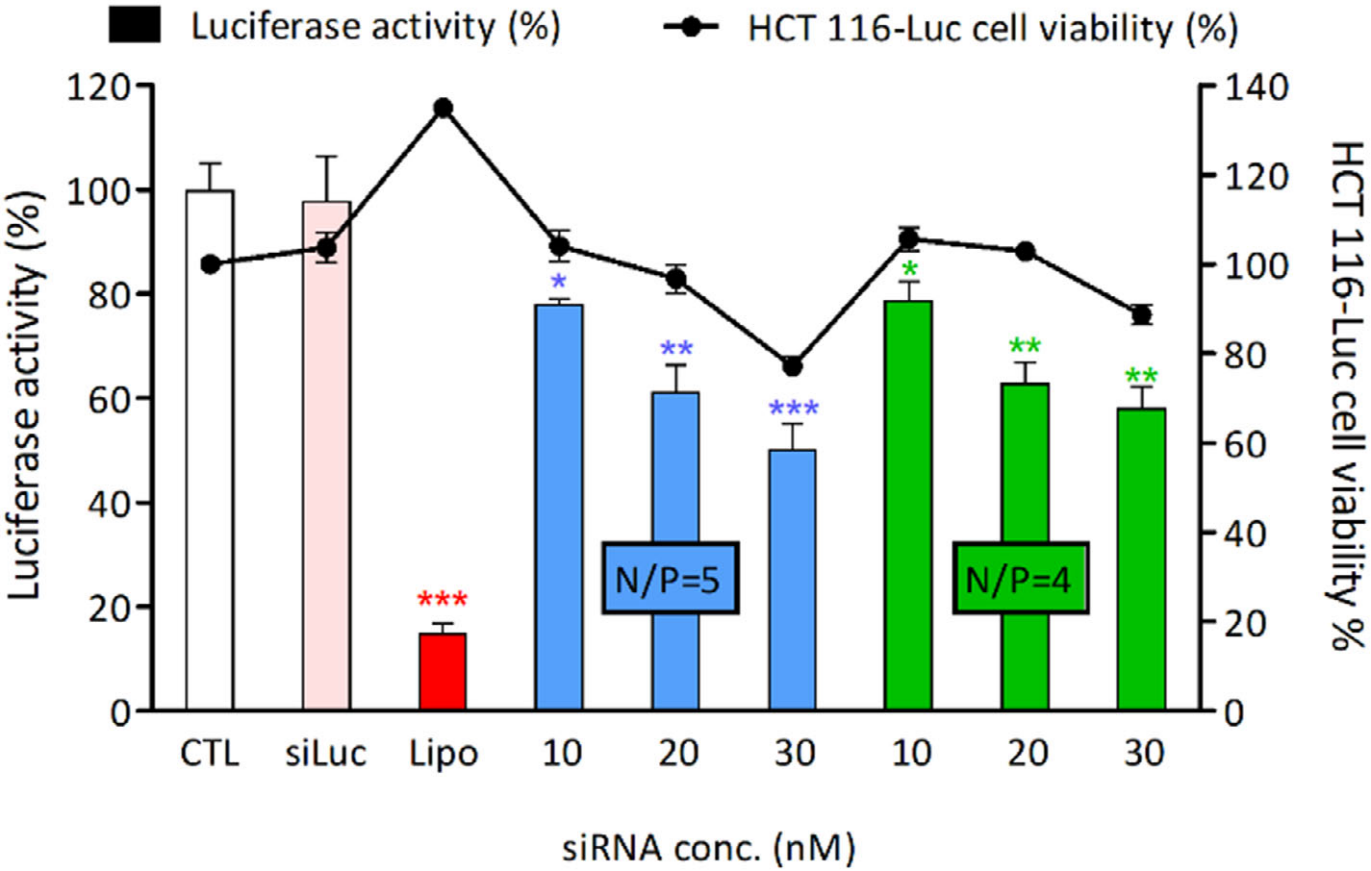
Knock‐down of luciferase activity on HCT 116‐luc cells by the ***Rac*‐1**–siLuc complexes at different N/P ratio and different siRNA doses. Lipofectamine RNAiMAX was used as a reference delivery vector and is noted as “Lipo.” Measurements were performed after 24 hr of incubation. The data are presented as mean ± SEM of three independent experiments. * Statistically significant difference from control condition (*p* < 0.01), ** Statistically significant difference (*p* < 0.001), *** Statistically significant difference (*p* < 0.0001).

Since no major differences were observed so far between ***M*‐1** and ***P*‐1**, it was decided, for simplicity, to select ***Rac*‐1** for the subsequent work. The efficacy of ***Rac*‐1** as a siRNA vector was assessed on the human colorectal cancer cells stably expressing luciferase activity (HCT 116‐Luc), using a siRNA against luciferase (siLuc). In order to mitigate the cytotoxicity, all experiments were carried out at maximum N/P = 5 and at the very low siRNA doses of 10, 20, and 30 nM so that, under these conditions, the concentration of ***Rac*‐1** is kept at the final concentrations of 2.1, 4.2, and 6.3 µM, respectively. The readout was also performed after 24 hr of incubation with ***Rac*‐1**–siLuc complexes. The results clearly showed a concentration‐dependent luciferase silencing effect with a decrease in luciferase activity of 22%, 39%, and 50% at siLuc doses of 10, 20, and 30 nM, respectively (**Figure** **4**). The cell viability of 77% at the maximum siLuc dose of 30 nM (i.e. 6.3 µM of ***Rac*‐1**) was found to be improved compared to the cell viability in the presence of the nanoassembly of ***Rac*‐1** alone without siRNA (45% at 5 µM of ***Rac*‐1**, Figure 3). We presume this can be the result of the siRNA‐loaded nanoparticles being less positively charged. Working at N/P = 4 further improves the cell viability from 77% to 89% but at the cost of a slightly decreased luciferase silencing activity that only decreased by 42% at the maximum siLuc dose of 30 nM (i.e. 5 µM of ***Rac*‐1**).

### 2.4 Cell Uptake and Subcellular Localization

**Figure 5:**
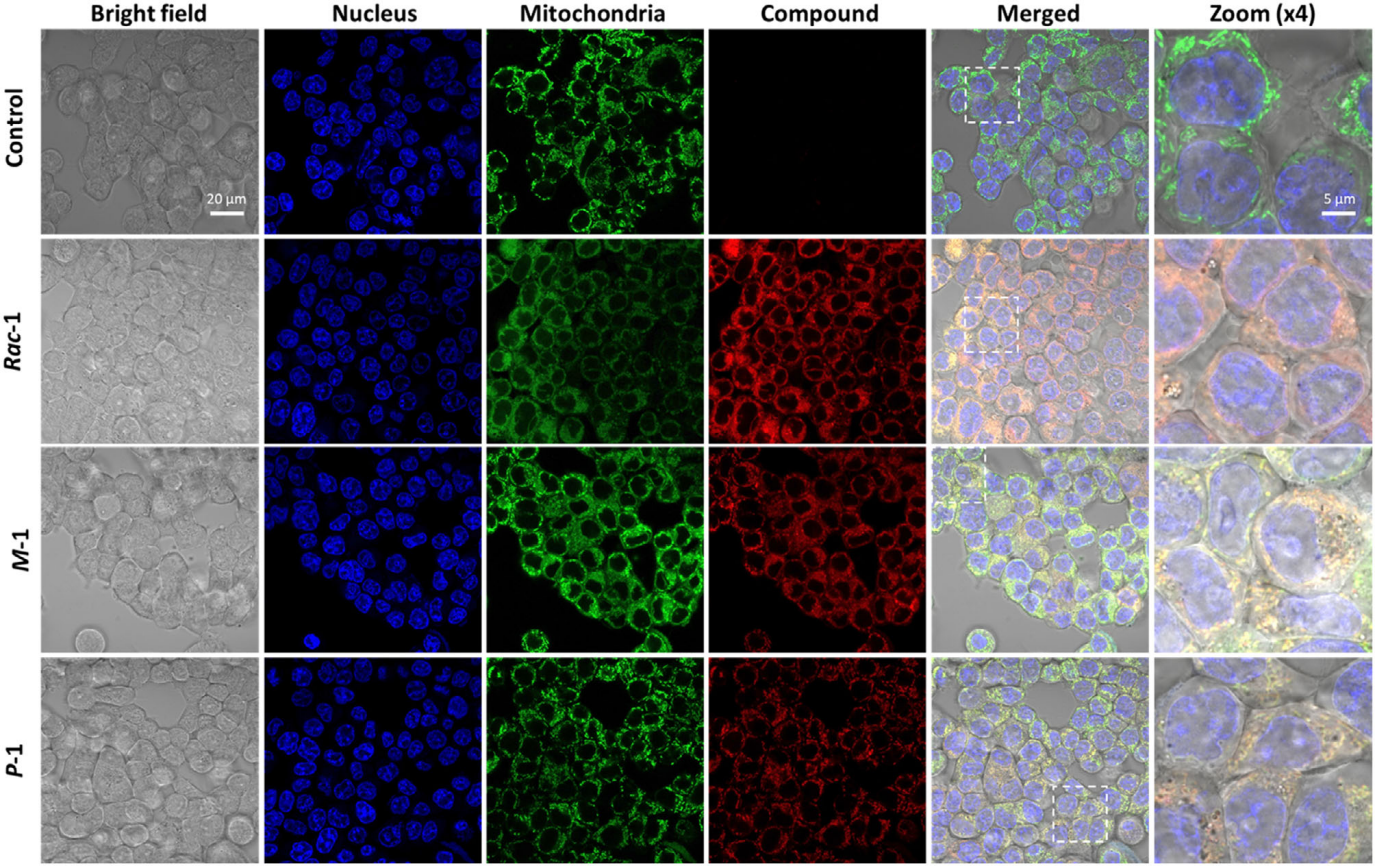
Subcellular localization study using mitoTracker and super‐resolution confocal microscopy imaging in living human colorectal cancer cells (HCT 116) treated (or not) with 2 µM of ***Rac*‐1**, ***M*‐1**, and ***P*‐1** for 3 hr of incubation.

The accumulation of the lipophilic cation **1** in mitochondria was confirmed on human colorectal cancer cells (HCT 116) by super‐resolution confocal microscopy imaging using MitoTracker to stain the mitochondria (**Figure** **5**). As the emergence of yellow color in the merged images is not enough to precisely express the degree of colocalization, a pixel intensity spatial correlation analysis using the ImageJ software was performed. Although ***M*‐1** seems to show a better cell uptake, the Pearson's coefficient values of 0.83, 0.84, and 0.81 were obtained for ***Rac*‐1**, ***M*‐1**, and ***P*‐1**, respectively, revealing an excellent colocalization in all cases.

For studying the subcellular localization of the ***Rac*‐1**–siRNA complexes in HCT 116, we selected an ATTO488‐labeled siRNA (siATTO 488, *λ*_ex_ = 488 nm, *λ*_em_ = 520 nm) in order to have a bioprobe orthogonal to the BioTracker 405 Blue Mitochondria dye (*λ*_ex_ = 405 nm, *λ*_em_ = 440 nm) and to the spectral features of ***Rac***‐**1** (*λ*_ex_ = 590 nm, *λ*_em_ = 650 nm).

**Figure 6:**
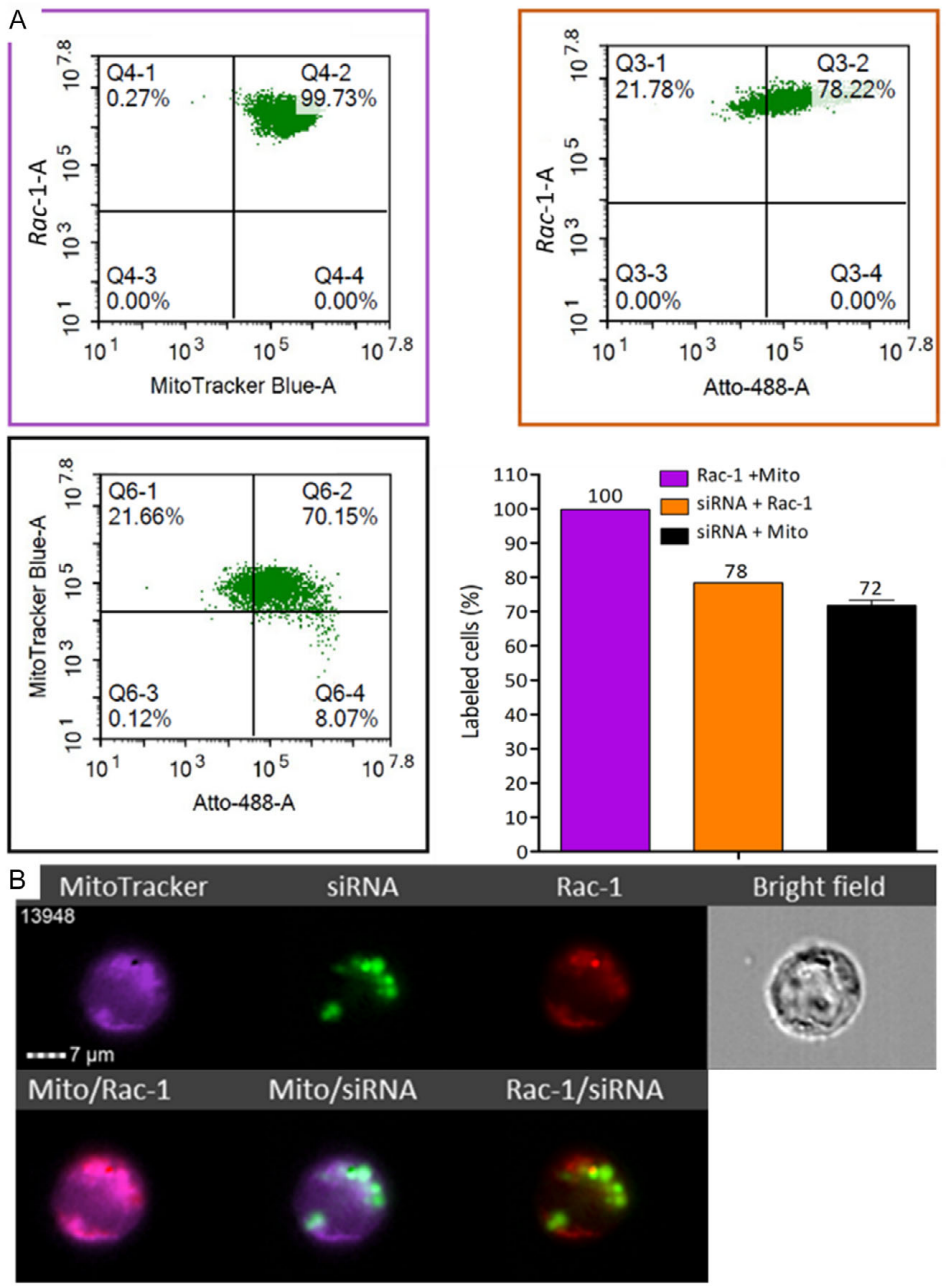
A) Flow cytometry analyses of ***Rac*‐1**–siATTO 488 (20 nM siATTO 488, N/P = 5) in HCT 116 cells after 24 hr of incubation. Cells were stained (or not) with 100 nM MitoTracker Blue, and fluorescence was quantified by flow cytometry. Data are presented as mean ± SEM of two independent experiments. B) Colocalization study using ImageStream flow cytometry after 5 hr of incubation.

Flow cytometry analyses revealed that ***Rac*‐1** and BioTracker 405 Blue are quantitatively cointernalized in HCT 116 cells (**Figure** **6**A). Also, around 70% of cells showed an uptake of both BioTracker 405 Blue Mitochondria dye and siATTO 488. All three dyes are therefore present in cells. Interestingly, a major fraction (78%) of ***Rac*‐1** was also cointernalized with siATTO 488 which suggests, since these experiments were performed at N/P = 5, that multiple ***Rac*‐1** are bound to siATTO 488 and internalized with it as a stable complex.

The subsequent use of ImageStream flow cytometry confirmed the subcellular colocalization (98% colocalization) of ***Rac*‐1** within mitochondria (Figure 6B) after 5 hr of incubation. It also showed a good colocalization of ***Rac*‐1** with siATTO 488 (48% colocalization), thus confirming the good stability of the ***Rac*‐1**–siATTO 488 complex in cells. Finally, it brought further support to the final good colocalization of siATTO 488 within mitochondria (42% colocalization).

### 2.5 Knockdown of Mitochondria‐Encoded Cytochrome c Oxidase Subunit 1 (MTCO1)

**Figure 7:**
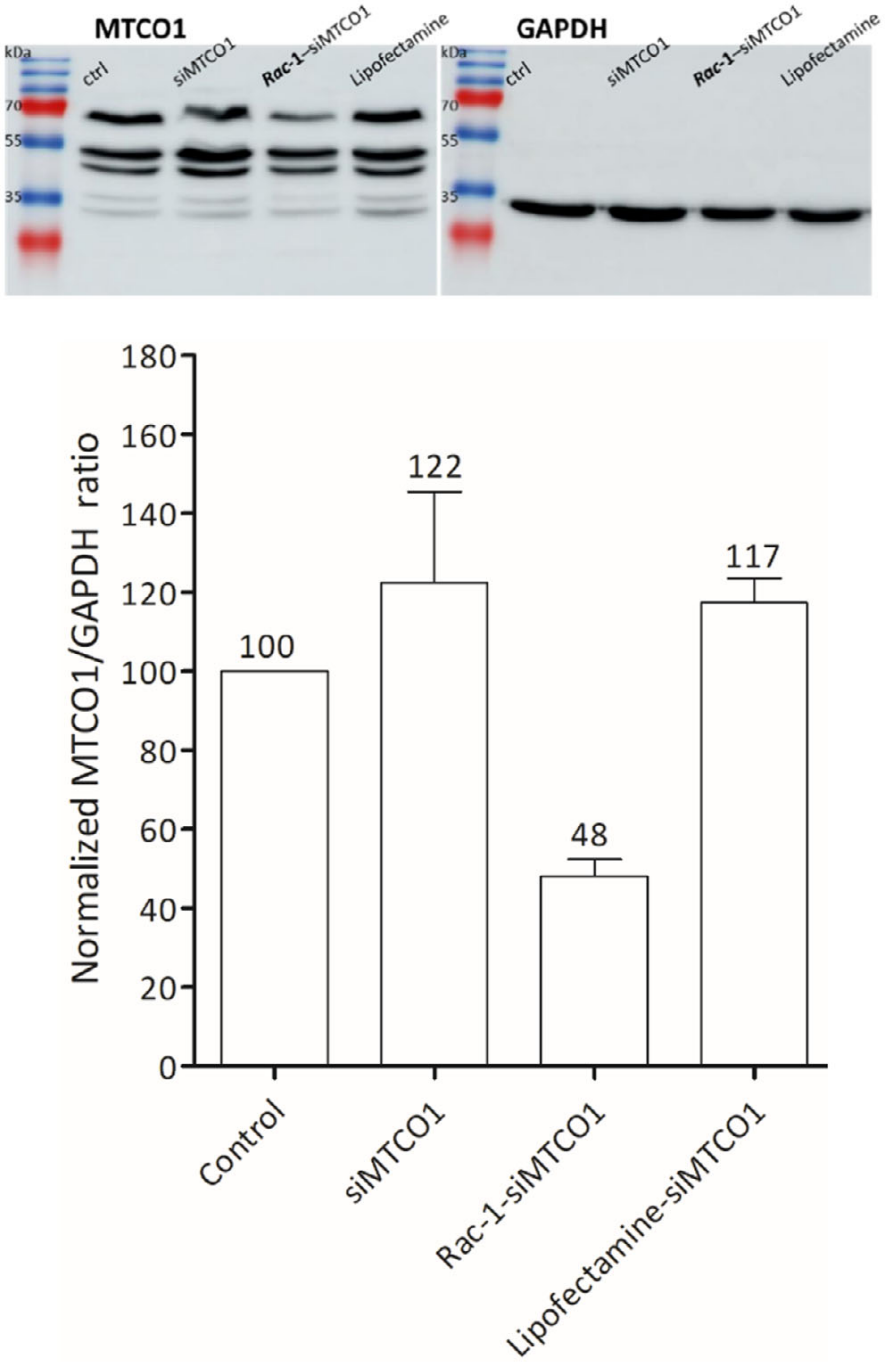
MTCO1 level in the HCT 116 cell lysates analyzed by western blotting: full gels (top) and corresponding histograms (bottom).

We tested the delivery of siMTCO1^[^ ^13^ ^]^ by the ***Rac*‐1** nanovector in order to silence the expression of the MTCO1 gene. Experiments were carried out using a 30 nM siMTCO1 dose and at N/P = 5. Quantification of total protein expression at 37 and 64 kDa was performed using densitometric analysis of western blot membranes.^[^ ^13^ ^,^ ^33^ ^,^ ^34^ ^]^ The values were compared to the glyceraldehyde‐3‐phosphate dehydrogenase (GAPDH) housekeeping protein to normalize protein loading.^[^ ^14^ ^]^ As shown in **Figure** **7**, the MTCO1 expression was significantly decreased to 48% only when using ***Rac*‐1**–siMTCO1, while no effect was observed by either siMTCO1 alone or lipofectamine, a transfection agent widely used in cationic lipid‐mediated gene transfer and best known for its remarkable endosomal escape propensity which results in an effective and diffuse delivery in the cytosol.^[^ ^35^ ^]^ This result therefore confirms that the ***Rac*‐1** nanovector is able to transport siRNA selectively to the mitochondria unlike other more conventional transfection agent.

## 3 Conclusion

We reported here that the small lipophilic chiral cation **1** is able to self‐assemble in the presence of siRNA, leading to the formation of nanoparticles characterized by DLS and *ζ* potential measurements. Complexation of siRNA was evidenced by electrophoretic mobility gel shift assays, revealing complete complexation at low N/P—this potency typically seen with cationic macromolecules is most likely the result of the amphiphilic self‐assembly of **1** into a nanovector inside which the siRNA will be bound.^[^ ^28^ ^,^ ^29^ ^]^ While minor differences between the enantiomers ***M*‐1** and ***P*‐1**, in line with previous results,^[^ ^23^ ^]^ were observed on siRNA complexation, the cell studies revealed that ***Rac*‐1** acts as an effective vector for siRNA, delivering siLuc in HCT 116 colorectal cancer cells with moderate activity (*ca.* 50% luciferase silencing) at a very low siRNA dose (30 nM), with good cell viability >70%. Yet, it remains less effective than lipofectamine for the knockdown of cytoplasmic proteins. However, the previously reported mitochondria‐targeting feature of the ***Rac*‐1** nanoassembly was found to extend to our siRNA‐loaded nanoparticles, as revealed by flow cytometry analyses showing a pronounced colocalization of ***Rac*‐1**, siATTO 488, and MitoTracker Blue. The knockdown of MTCO1 was demonstrated by western blotting following the delivery of siMTCO1 by ***Rac*‐1**, which now performs much better than the gold standard lipofectamine. Thus, this work provides a proof‐of‐concept that a small molecule can effectively deliver siRNA in targeted organelles to trigger a silencing effect. The specific ability of ***Rac*‐1** to target mitochondria and to transport RNA in this organelle can open up perspectives in modulating the cell fate by silencing the expression of mitochondrial genome.^[^ ^36^ ^]^

## 4 Experimental Section

4.1

4.1.1

##### Agarose Gel Shift Assay

A fixed concentration of control siRNA (2 µM) was added to increasing concentrations (42.5–840 µM) of racemic compounds, in order to reach N/P ratio ranging from 0.5 to 10. The complex solutions were prepared at pH 7.2 and incubated at 25 °C for 30 min. The complex solutions were then mixed with 6X DNA Loading Dye (ThermoScientific, France). Electrophoresis was carried out on a 2% (w/v) agarose gel containing GelRed nucleic acid gel stain (Interchim, France). The gel was run in 0.5x Tris−Borate−EDTA buffer (Euromedex, France) for 40 min at 50 V. A directLoad PCR 100 bp low Ladder (Sigma–Aldrich, USA) was used. The siRNA bands were visualized by TFX‐20 M model‐UV transilluminator (Vilber Lourmat, France), and gel photographs were obtained with a smartphone camera. The high binding capacity between siRNA and racemic compounds is expressed by the disappearance of siRNA bands on the gel.

##### Physicochemical Characterization

Different siRNA‐***Rac‐1*** complexes were prepared in 5% glucose solution at N/P ratios of 5, 10, and 20. Dynamic light scattering (DLS) and *ζ* potential measurements were obtained by using Zetasizer Nano ZS (Malvern, United Kingdom). Six measurements were made with 12 runs for each.

##### Cell Lines

Human colorectal cancer cells (HCT 116) were purchased from ATCC. The HCT 116‐Luc cell line was obtained from IRCM Cell Culture Unit (Montpellier, France). Cells were maintained in McCoy 5A modified medium (Gibco, France) supplemented with 10% fetal bovine serum (FBS) and 0.5% gentamycin (Gibco, France). All cell lines were incubated at 37 °C in a humidified atmosphere with 5% CO_2_.

##### Cytotoxicity

HCT 116 cells were seeded in 96‐well plate at a density of 1 × 10^4^ cells *per* well (200 µL) in their respective medium. Twenty‐four hours after seeding, cells were treated with different concentrations of ***Rac*‐1**, ***M*‐1**, and ***P*‐1** and left to incubate for 24 hr and 48 hr. Cells treated with the vehicle were considered as a control. Cell viability was measured using 4,5‐dimethylthiazol‐2‐yl)−2,5‐diphenyltetrazolium bromide (MTT) (Sigma–Aldrich, USA) assay as previously described.^[^ ^37^ ^]^ The percentage of viable cells was calculated according to the following equation: viability (%) = Absorbance_test_/ Absorbance_control_*100. The experiments were performed in triplicates.

##### siRNA Delivery and Cell Luciferase Assay

HCT 116‐Luc cells were seeded in 96‐well white plate, PS, F‐bottom, µCLEAR (Greiner Bio‐one, Germany) at a density of 1 × 10^4^ cells *per* well (200 µL) in their respective medium supplemented with heat‐inactivated FBS (FBS^−^) and gentamycin. Twenty‐four hours after seeding, cells were treated (or not) with ***Rac*‐1**–siLuc complexes for 4 h. Complexes were freshly prepared in serum‐free cell culture medium for 30 min at 25 °C using different concentration of siLuc (10, 20, 30 nM) and ***Rac‐1*** (1.68, 3.36 ,and 5 µM, respectively, to achieve N/P = 4 and 2.1, 4.21, and 6.32 µM, respectively, to achieve N/P = 5). Cells treated with lipofectamine RNAiMAX (Invitrogen, USA) were considered as positive control. After 4 h of incubation, FBS^−^ was added in each well to reach a final concentration of 4%. Twenty‐four hours after incubation, luciferase activity was evaluated by adding D‐luciferin (PerkinElmer, USA) at a final concentration of 318 µg mL^−1^. Cell luminescence was measured 10 min after using Thermo Scientific Varioskan LUX multimode microplate reader. Luciferase activity was corrected according to the total number of living cells in each well determined by MTT assay. Results are expressed as a percentage of luminescence activity of treated cells compared to the control non‐treated cells (set as 100%). The experiment was repeated three times.

##### Subcellular Localization Study Using Super‐Resolution Microscopy

HCT 116 cells were seeded in µ‐slide 8 well (Ibidi GmbH, Germany) at a density of 15 × 10^4^ cells. One day after seeding, cells were treated with 2 µM (final concentration in cell culture medium) of ***Rac‐1, M*‐1**, and ***P*‐1** for 3 hr. Forty‐five minutes before the end of incubation, cells were treated with 100 nM (final concentration in cell culture medium) of MitoTracker green FM (Invitrogen, USA). Fifteen minutes before the end of incubation, cells were treated with Hoechst 33,342 (Invitrogen, USA) at a final concentration of 10 µg mL^−1^. Cells were washed three times with culture medium before observation with super‐resolution confocal fluorescent microscope LSM880 Airyscan (Carl Zeiss, France) at excitation wavelengths of 488 nm for MitoTracker, 561 nm for ***Rac‐1***, and 405 nm for Hoechst, using a high magnification (63x). After acquisition, images were processed using Airyscan processing. The Pearson's coefficient was calculated using ImageJ software.

##### Colocalization Study of Rac‐1/siRNA Complex Using Flow Cytometry

HCT 116 cells were seeded in a 12‐well plate at a density of 2 × 10^5^ cells. Twenty‐four hours after seeding, cells were treated (or not) with complexes of ***Rac*‐1**–siATTO 488 at N/P = 5 (20 nM of siRNA and 4.2 µM ***Rac‐1***). After 4 h of incubation, FBS^−^ was added in each well to reach a final concentration of 4%, and the cells were then left to incubate for 24 hr or 5 hr. Forty‐five minutes before the end of incubation, cells were treated (or not) with 100 nM of BioTracker 405 Blue Mitochondria Dye (EMD Millipore Corp., USA). After, cells were washed three times with phosphate‐buffered saline (PBS), trypsinized, centrifuged, and then suspended in PBS containing 1 mM EDTA. Flow cytometric analysis was performed using Agilent NovoCyte flow cytometer, and the data were analyzed by the NovoExpress software (ACEA Biosciences, Inc.). The analysis was carried out in 20,000 events. The experiment was repeated two times in duplicate. Samples were also analyzed by ImageStreamX MkII system using 60x magnification, and the data were analyzed by the IDEAS software (cytek biosciences, USA).

##### Knockdown of Mitochondria‐Encoded Cytochrome c Oxidase Subunit 1 (MTCO1)

HCT 116 cells were seeded in 12‐well plate at a density of 35 × 10^4^ cells. Twenty‐four hours after seeding, cells were treated with siMTCO1 (30 nM) alone, ***Rac*‐1** (6.5 μM)/siMTCO1 (30 nM) complex, and Lipofectamine/siMTCO1. After 4 hr of incubation, FBS^−^ was added in each well to reach a final concentration of 4% and cells were left to incubate for 24 hr. Cells washed two times with PBS, trypsinized, and collected by centrifugation at 1300 rpm for 5 min followed by further washing with PBS. Cells were lysed using lysis buffer and three freeze–thaw cycles followed by centrifugation at 14,000 rpm for 15 min. The concentration of the extracted protein in the obtained supernatant was determined using Bradford reagent (Abcam, UK). The knockdown effect of siMTCO1 was determined by Western blot analysis. Thirty micrograms of denaturated proteins were resolved on 15% SDS‐PAGE; after migration at 140 mV for 2 hr, the gel was transferred onto PVDF membrane then after blocking, MTCO1 was detected using MTCO1 polyclonal antibody (Invitrogen, USA) at 1:500 dilution followed by anti‐rabbit HRP‐conjugated antibody (1:7500, Jackson ImmunoResearch). Membranes were revealed by ECL detection reagents (Amersham, Cytiva, UK). GAPDH was used as a housekeeping protein control. After membrane stripping and blocking, the membrane was probed with GAPDH monoclonal antibody (Proteintech, UK) at dilution 1:50,000 followed by anti‐mouse HRP‐conjugated antibody (1:10,000, Jackson ImmunoResearch) and ECL detection. The obtained bands were quantified using the ImageJ software. The MTCO1 bands were normalized to the bands of GAPDH, and the percentage of MTCO1 protein expression was calculated by considering control bands as 100%. The experiment was repeated three times.

##### Statistical Analysis

Results are presented as the mean ± standard error of the mean (SEM). Statistical analysis was performed using GraphPad Prism. The comparison between groups was analyzed with Student's t‐test. Differences were considered statistically significant when *p* values were less than 0.01 (*p* < 0.01). The level of significance was defined at *p < 0.01, **p < 0.001, and ***p < 0.0001.

## Conflict of Interest

The authors declare no conflict of interest.

## Data Availability

The data that support the findings of this study are available from the corresponding author upon reasonable request.
